# One-Year Follow-Up of Natural Killer Cell Activity in Multiple Myeloma Patients Treated With Adjuvant Lenalidomide Therapy

**DOI:** 10.3389/fimmu.2018.00704

**Published:** 2018-04-13

**Authors:** Laurie Besson, Emily Charrier, Lionel Karlin, Omran Allatif, Antoine Marçais, Paul Rouzaire, Lucie Belmont, Michel Attal, Christine Lombard, Gilles Salles, Thierry Walzer, Sébastien Viel

**Affiliations:** ^1^CIRI, Centre International de Recherche en Infectiologie—International Center for Infectiology Research, Lyon, France; ^2^INSERM, U1111, Lyon, France; ^3^Ecole Normale Supérieure de Lyon, Lyon, France; ^4^Université Lyon 1, Lyon, France; ^5^CNRS, UMR5308, Lyon, France; ^6^Laboratoire d’Immunologie, Hospices Civils de Lyon, Centre Hospitalier Lyon Sud, Pierre-Bénite, France; ^7^Hospices Civils de Lyon, Centre Hospitalier Lyon Sud, Service d’Hematologie, Pierre-Benite, Universite Claude Bernard Lyon 1, Lyon, France; ^8^Service d’Immunologie, CHU de Clermont-Ferrand, équipe ERTICa EA4677, Université d’Auvergne, Clermont-Ferrand, France; ^9^Institut Universitaire du Cancer de Toulouse-Oncopole, Toulouse, France

**Keywords:** natural killer cells, lenalidomide, immunomodulatory drugs, immunomonitoring, innate immunity, multiple myeloma

## Abstract

Multiple myeloma (MM) is a proliferation of tumoral plasma B cells that is still incurable. Natural killer (NK) cells can recognize and kill MM cells *in vitro* and can limit MM growth *in vivo*. Previous reports have shown that NK cell function is impaired during MM progression and suggested that treatment with immunomodulatory drugs (IMIDs) such as lenalidomide (LEN) could enhance it. However, the effects of IMIDs on NK cells have been tested mostly *in vitro* or in preclinical models and supporting evidence of their effect *in vivo* in patients is lacking. Here, we monitored NK cell activity in blood samples from 10 MM patients starting after frontline induction chemotherapy (CTX) consisting either of association of bortezomib–lenalidomide–dexamethasone (Velcade Revlimid Dexamethasone) or autologous stem-cell transplantation (SCT). We also monitored NK cell activity longitudinally each month during 1 year, after maintenance therapy with LEN. Following frontline chemotherapy, peripheral NK cells displayed a very immature phenotype and retained poor reactivity toward target cells *ex vivo*. Upon maintenance treatment with LEN, we observed a progressive normalization of NK cell maturation, likely caused by discontinuation of chemotherapy. However, LEN treatment neither activated NK cells nor improved their capacity to degranulate or to secrete IFN-γ or MIP1-β following stimulation with MHC-I-deficient or antibody-coated target cells. Upon LEN discontinuation, there was no reduction of NK cell effector function either. These results caution against the use of LEN as single therapy to improve NK cell activity in patients with cancer and call for more preclinical assessments of the potential of IMIDs in NK cell activation.

## Introduction

Multiple myeloma (MM) is a genetically heterogeneous disease characterized by a proliferation of tumoral plasma B cells (1). MM is among the most frequent hematologic malignancies and induces anemia, bone lesions, and renal dysfunction. Despite the emergence of several novel dedicated therapies, MM remains of poor prognosis, because most patients become refractory to treatments (2). MM frequently progresses from a premalignant stage called monoclonal gammopathy of undetermined significance. Upon diagnosis, patients present either smoldering (or indolent) asymptomatic MM or symptomatic MM with organ damages that require immediate treatment. Efforts are underway to understand genetic and other factors that influence progression of indolent to active MM and to find treatments that could prevent or delay this progression (1).

Cytotoxic lymphocytes such as natural killer (NK), and CD8 T cells are important actors of tumor immune surveillance. NK cells mediate various antitumor functions, including granule-dependent cytotoxicity, secretion of cytokines like TNF-α and IFN-γ, as well as secretion of CCL3-5 chemokines, that have distinct roles during immune responses (3). NK cells recognize tumor cells through an arsenal of activating or inhibitory receptors. They can also recognize and kill antibody-coated target cells through CD16, the low-affinity activating receptor for the Fc fragment of immunoglobulins G. This process is called antibody dependent cell-mediated cytotoxicity (ADCC) (4) and is supposed to be one of the mechanisms of action of many therapeutic monoclonal antibodies (5).

Natural killer cells have long been shown to recognize and kill MM cells *in vitro*. HLA and activating receptors such as NKG2D, DNAM1, and NKp46 are important for the recognition of MM cells by NK cells (6–8). However, as for many tumors, other articles have suggested a gradual alteration of peripheral NK cell activity during MM progression [reviewed in Ref. (9)].

Lenalidomide (LEN) is part of the immunomodulatory drugs (iMIDs) and is a synthetic derivative of thalidomide currently approved by regulatory agencies for the treatment of MM, in combination with dexamethasone. LEN presumably targets the ubiquitin ligase Cereblon (CRBN), and CRBN is required for LEN antitumor function (10). The mechanisms of LEN actions in MM include direct antitumor effects, effects on tumor stroma and angiogenesis, and a positive effect on the antitumor immune response (11). LEN affects many cell types and in particular enhances the antitumor functions of NK and NKT cells (12). This could be due to several mechanisms. First, LEN could suppress the production of inhibitory cytokines and promote the production of IL-2, an NK-cell growth factor, by T cells (13). The latter effect could be the result of induced degradation of Ikaros and Aiolos, two transcription factors repressing IL-2 production in T cells (14). Moreover, LEN can enhance the expression of the NKG2D and DNAM-1 activating receptor ligands MICA and PVR/CD155 also through the downregulation of Ikaros and Aiolos (15). LEN could also operate directly *in vitro* as an article demonstrated that LEN enhanced cytotoxicity and IFN-γ production by purified NK cells stimulated through various receptors, in the presence of stimulatory concentrations of IL-2 (16). The proposed mechanism involves nanometer-scale rearrangement of the actin cytoskeleton at the immune synapse even though LEN targets were not identified in this context. Importantly, in this study, LEN alone had limited activity (16), thus showing that indirect effects on IL-2 production are mandatory for the improvement of NK cell cytotoxicity. Despite accumulating evidence of the stimulatory activity of LEN on immune cells *in vitro* or in mouse preclinical models, very few studies have addressed the effect of LEN on immune cells in LEN-treated MM patients. One longitudinal study did not report any effect of LEN on NKT cells in a small number of patients (17). Another one reported weak signs of NK cell activation 1 month after the beginning of LEN as maintenance therapy, but the interpretation of the results was complicated by the prior allogenic stem-cell transplantation (SCT) of all patients and the discontinuation of immunosuppressive therapy used to reduce GVHD at the time of LEN treatment (18). Thus, a stimulatory effect of LEN on NK cell activity in human *in vivo* remains to be formally proven. To address this point, we monitored NK cells in patients with MM treated only with LEN as maintenance chemotherapy.

## Materials and Methods

### Patients and Samples

Patients were recruited in the context of the IFM/DFCI 2009 trial (#NCT01191060) and followed in the Hospital Lyon Sud. Patients under 65 years old with newly diagnosed symptomatic MM were randomized to receive, after frontline induction regimen with three cycles of bortezomib–lenalidomide–dexamethasone (VRD for Velcade/Revlimid/Dexamethasone), either SCT conditioned with high dose of Melphalan, followed by a two-cycle VRD consolidation, or five additional VRD cycles without high dose therapy. The two arms then received 1 year maintenance with single agent LEN. Patients’ characteristics are summarized in Table 1 and results of the clinical trial were recently published (19).

**Table 1:** Clinical and biological characteristics of LEN-treated patients.

	Group A (VRD) (*n* = 7)	Group B (SCT) (*n* = 3)
Male/female	5/2	2/1
Age (range)	59 (50–67)	60 (48–65)
**Myeloma subtype**		
IgG	5	2
IgA	3	1
Median immunoglobulin peak levels (range) (g/L)	29 (10.1–75.8)	19.8 (1.5–43.4)
Median β2 microglobulin levels (range) (mg/L)	3.1 (2.7–13.6)	2 (1.3–2.3)
**LEN dose**		
Median no. of lenalidomide (LEN) cycles	13	12
10 mg/day	5	1
15 mg/day	2	2
**Patient outcome**		
Remission	3	0
Relapse	4	2
Death	0	1

*VRD, Velcade Revlimid Dexamethasone, SCT, stem-cell transplantation*.

The clinical trial protocol was initially approved by the institutional ethics committee at the coordinating center (Purpan Hospital, Toulouse, France). For our study, patients provided an additional written informed consent in accordance with the Declaration of Helsinki.

Blood of age- and sex-matched healthy volunteers came from blood donors of the Etablissement Français du Sang Rhône-Alpes, who had given their written informed consent according to the Institutional Research Protection Guidelines.

All heparinized blood samples were received and processed within 4 h after collection. Whole blood was stained for phenotypic markers, and PBMC were obtained using ficoll. PBMC were stored at 4°C overnight and stimulated the next day. In most cases, one to three blood samples from patients were received and processed per day. To avoid any bias in subsequent comparisons, blood samples from healthy donors were also processed by groups of three samples maximum.

### Phenotypic Analysis of NK Cells and Absolute Counts

We stained fresh whole blood with four different antibody panels covering the whole range of markers analyzed (100 µL per staining, Table 2). Intracellular stainings were performed after staining for cell surface markers using Cytofix/Cytoperm from BD biosciences. Tubes were run on a Beckman-Coulter Navios instrument. To allow comparisons between experiments, we always used the same FACS settings. We performed routine testing of the instrument using Rainbow calibration particles (Spherotech) using these FACS settings, to check for laser parameters, and if appropriate, PMT were slightly modulated to compensate for laser variations. Whenever possible, a minimum of 3000 NK cells were acquired for each sample. Data were analyzed using Kaluza (Beckman Coulter) software. NK cells were defined as CD3/4/14/19^−^ CD56^+^ cells (Figure S1A in Supplementary Material). Antibodies used for the identification of NK cells were purchased from Beckman Coulter: CD3 APC-AF750 (UCHT1), CD4 ECD (SFCI12T4D11), CD14 ECD (RMO52), CD19 ECD (J3-119), and CD56 APC (N901). Figure S1 in Supplementary Material shows representative FACS plot of Surface receptors (Figure S1B in Supplementary Material) and Activation markers (Figure S1C in Supplementary Material) analyzed. References of the antibodies used are listed in Table 2. Absolute lymphocyte subsets counts were determined by multiplying the percentage of the subset among lymphocytes by the absolute lymphocyte counts determined using the hematological analyzer SYSMEX (Roche).

**Table 2:** Natural killer cell surface or intracellular parameters analyzed.

	Clone	Fluorochrome	Source
**Antitumor function**			
CD107a	eBioH4A3	FITC	eBiosciences
TNF-α	MAb11	Pe-Cy7	Biolegend
IFN-γ	4S.B3	PE	eBiosciences
MIP1-β	D21-1351	V450	BD
**Activation markers**			
CD69	TP1.55.3	PE	Beckman
Granzyme B	GB11	FITC	Biolegend
NKG2C	134591	AF488	R&D Systems
NKG7	2G9A10F5	PE	Beckman
Perforin	deltaG9	PerCp-ef710	eBiosciences
**Surface receptors**			
DNAM-1	TX25	FITC	Biolegend
CX3CR1	2A9-1	PE-Cy7	Biolegend
KIR2DL2	DX27	FITC	Biolegend
KIR2DL1	HP-MA4	PE-CY7	eBiosciences
KIR3DL1	DX9	AF700	Biolegend
CD16	3G8	Pac Blue	Biolegend
CD57	TB01	eF450	eBiosciences
CD161	HP-3G10	PerCp-Cy 5.5	eBiosciences
2B4	C1.7	PerCp-Cy 5.5	Biolegend
NKp30	P30-15	PE	Biolegend
NKG2D	1D11	PE-Cy7	Biolegend
NKp46	9E2	BV421	Biolegend
CD94	HP-3D9	PerCp-Cy 5.5	BD

### Functional Analysis of NK Cells

PBMC were co-cultured for 4 h at a 1:1 ratio with K562 cells or Granta B lymphoma cells (ATCC) or cultured alone (“no stim” condition). Granta cells were incubated with anti-CD20 antibody (Rituximab, Hoffman Laroche) for 30 min at 4°C before stimulation. Golgi-stop (BD Biosciences) was added after 1 h of culture. Cells were stained for surface markers (CD3/4/14/19/56/CD107a) before staining for intracellular cytokines (IFN-γ, MIP1-β, and TNF-α). Figure S1D in Supplementary Material shows representative data of functional analyses. References of the antibodies used are listed in Table 2. During the setup phase of this study, we noticed a significant drop in NK cell responses when stimulating PBMC with overgrown K562 or Granta cells, or over successive passages. For this reason, we made a stock of frozen K562 and Granta aliquots at the beginning of the project and never kept them in culture for more than 2 months. Moreover, cell stimulations were always performed during the next 2 days after target cell passage. To analyze polyfunctionality and determine the frequency of NK cells able to combine two (for example, CD107 exposition + production of IFN-γ or MIP-1β) or three functions (CD107a exposition + production of both IFN-γ and MIP-1β), we used FlowJo (Treestar) and the “Boolean gates” function. As TNF-α production was negligible, we focused on CD107a exposition and IFN-γ and MIP1-β production.

### Statistical Analyses

Statistical analysis was performed using the R computing environment, R version 3.2.3 (20). Heatmap figures were produced with the gplots package using the “heatmap.2” function. See the figure legends for details about when logistic regression, linear mixed-effects models were used.

## Results

Patients were enrolled in a clinical trial conducted by the Intergroupe Francophone du Myélome (IFM group, protocol IFM/DFCI 2009, see Materials and Methods). The induction/consolidation CTX consisted in either eight cycles of bortezomib, dexamethasone, and LEN (VRD for Velcade/Revlimid/Dexamethasone) or in autologous SCT, followed by two VRD cycles (Table 1; see Materials and Methods) (21). In both arms of the study, induction and consolidation CTX were followed by maintenance treatment with LEN as single agent for 1 year, starting 1 month after the last VRD cycle (Table 1; see Materials and Methods). Blood samples were obtained from patients 1 month after the end of the last VRD cycle and during treatment with LEN, on a monthly basis over the 1-year treatment. We also monitored NK cell phenotype and function after the end of the last LEN cycle (on average 6 months after the last LEN cycle).

To analyze the phenotype of fresh blood NK cells, we measured, by flow cytometry, their expression of activating and inhibitory receptors, activation markers, and molecules associated with NK cell maturation and cytotoxicity (Table 2). We also performed a functional analysis. Degranulation as measured by CD107a exposure and IFN-γ, TNF-α, and MIP1-β secretion were measured upon a 4-h *ex vivo* culture without stimulus (no stim) or in the presence of K562 cells or Granta B cells coated with rituximab anti-CD20 antibody, to measure natural cytotoxicity and ADCC, respectively. Two types of measurements were performed: frequency of NK cells positive for each functional marker (CD107a, IFN-γ, and MIP1-β) in the K562, Granta or medium condition and frequency of polyfunctional NK cells (two or three functions simultaneously, only for K562 and Granta culture conditions, see Materials and Methods).

### Induction CTX Reduces NK Cell Maturation

**Figure 1:**
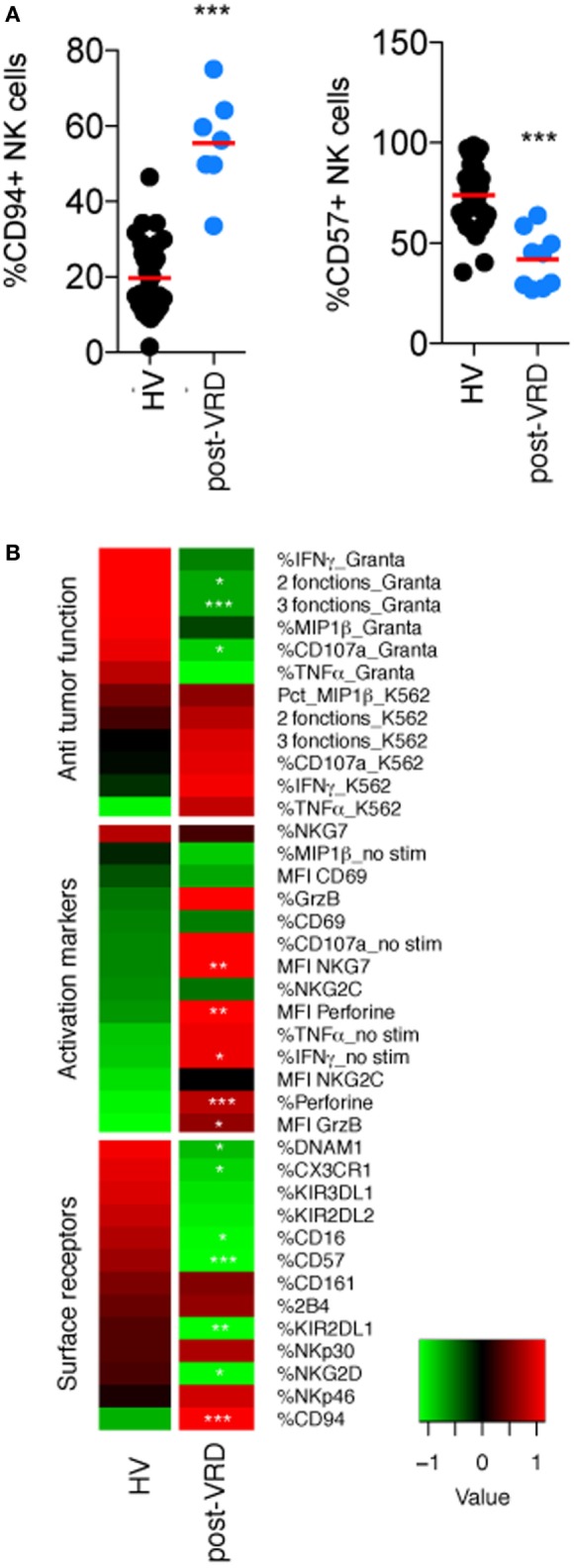
Induction CTX impairs natural killer (NK) cell maturation and ADCC functions. Flow cytometry analysis of the indicated parameters in peripheral NK cells from HV and multiple myeloma patients after induction/consolidation CTX (post-Velcade Revlimid Dexamethasone). **(A,B)** Parameters were clustered in functional categories “antitumor function,” “activation markers,” and “cell surface receptors” as labeled and displayed as heatmaps (black dots are patients who received Velcade Revlimid Dexamethasone and blue dots are patients who received stem-cell transplantation). Stars summarize significance level of the *p*-values adjusted for multiple testing by the Benjamini–Hochberg method. These *p*-values were obtained using logistic regression models, which compare each patient group with the HD group. Significance codes: 0 “***” 0.001 “**” 0.01 “*” 0.05 “ ” 1.

We first observed that the induction/consolidation CTX had a profound impact on NK cell maturation, as assessed by the percentage of NK cells expressing CD16, CD94, and CD57 (22) (Figures 1A,B; Figure S2 in Supplementary Material), which probably reflects the elimination of most mature NK cells during CTX and the progressive reappearance of neo-developed NK cells with an immature CD94^+^ CD57^−^ phenotype. The skewed NK cell maturation in the CTX group could also explain the reduced expression of various other receptors associated with NK cell maturation such as KIRs, DNAM1, and CX3CR1 (Figure 1B; Figure S2 in Supplementary Material). After induction CTX, NK cells phenotype was also characterized by a higher expression of activation markers such as Granzyme B and Perforin (Figure 1B; Figure S2 in Supplementary Material). In terms of function, NK cell reactivity to K562 cells, which is known to be dependent on NKp30 (23) was not significantly modified by CTX as far as the expression of NKp30 itself. On the other hand, NK cell reactivity to Granta cells was severely reduced (Figure 1B; Figure S2 in Supplementary Material). This effect on ADCC could be due to the reduced percentage of CD16 expressing cells among neo-developed immature NK cells (Figure 1B; Figure S2 in Supplementary Material).

Thus, induction/consolidation CTX has a profound effect on the peripheral NK cell compartment, altering overall maturation, and consequently narrowing the repertoire of NK cell receptors expressed and their range of functions.

### Longitudinal Study of the Impact of LEN on NK Cell Phenotype and Function

Lenalidomide is often cited as an NK cell-stimulating agent, based on *in vitro* data or on experiments in mouse models. However, evidence that LEN stimulates NK cell activity in patients is still lacking. To address this point, we took advantage of the protocol IFM/DFCI 2009. In both arms of the study, induction and consolidation CTX were indeed followed by maintenance treatment with LEN as single agent for 1 year, starting 1 month after the last VRD cycle (Table 1; Materials and Methods). We monitored NK cell phenotype and function before and during treatment with LEN, on a monthly basis over the 1-year treatment. We also monitored NK cell phenotype and function after the end of the last LEN cycle (on average 6 months after the last LEN cycle).

**Figure 2:**
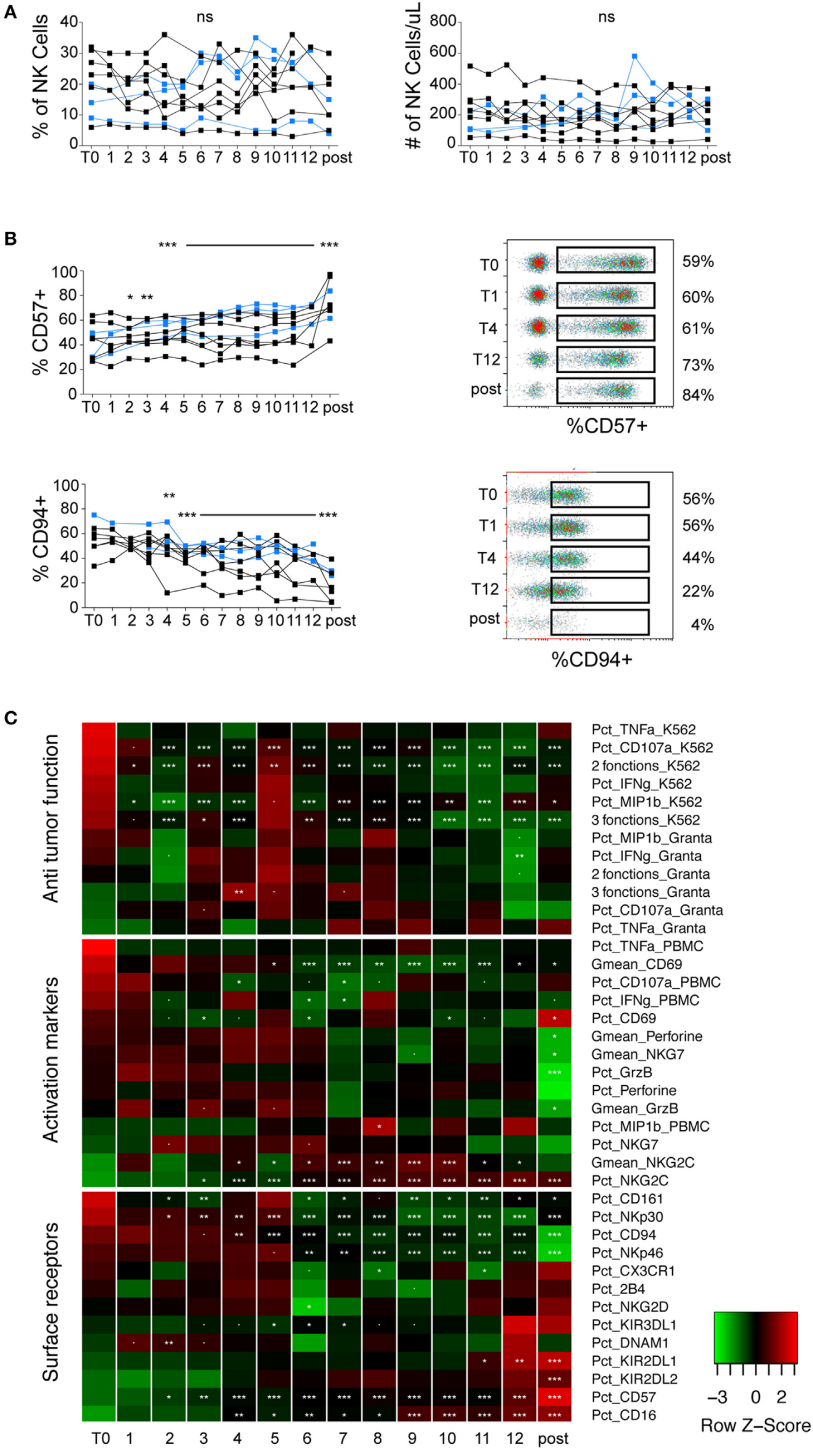
LEN treatment neither activates NK cells nor improves their effector functions. Flow cytometry analysis of the indicated parameters in NK cells from patients monitored at different time-points before, during, or after LEN therapy. Data were obtained and analyzed as indicated in Figure 1. **(A)** Charts of the percentages and absolute counts of NK cells. **(B)** Charts of the percentages of CD57 and CD94 positive cells within gated NK cells. Each line corresponds to one patient (black dots are patients who received VRD, blue dots are patients who received SCT). **(C)** Parameters were clustered in functional categories “anti-tumor function”, “activation markers,” and “cell surface receptors” as labeled and displayed in heatmaps. Stars summarize significance level of the p-values obtained using linear mixed effect models analyzing the effect of LEN on each parameter, compared to the pre-treatment time-point (T0, corresponding to 3 weeks after CTX). Our analysis takes into account the fact that data are paired over time-points. Significance codes: 0 “***” 0.001 “**” 0.01 “*” 0.05 “.” 0.1 “ ” 1.

Overall, we did not observe any effect of LEN on NK cells numbers (Figure 2A). The most striking effect we observed over the treatment was a gradual increase in NK cell maturation status, as shown by a decrease in CD94 or NKp30 positive cells (markers of immature NK cells) and an increase in CD57 or CD16 positive cells (markers of mature NK cells) (Figures 2B,C; Figure S3 in Supplementary Material). Changes in KIR2DL1 and NKp46 could also fit within this scheme (Figure 2C; Figure S3C in Supplementary Material). This effect was, however, likely not due to LEN treatment but rather to the progressive return to immune homeostasis following induction/consolidation CTX or SCT (blue dots in Figure 2B; Figure S3 in Supplementary Material). Indeed, the effect was not immediate following the beginning of LEN treatment and persisted after LEN discontinuation, e.g., KIR2DL1, NKp30, NKp46 (Figure 2C; Figure S3C in Supplementary Material), Granzyme B, and Perforin (Figure 2C; Figure S3B in Supplementary Material). Regarding activation markers, we noted a progressive increase in the percentage and MFI of NKG2C expression during LEN treatment (Figure 2C; Figure S3B in Supplementary Material). This effect was, however, not associated with an increase in any other activation marker, and persisted after LEN discontinuation, suggesting that it was not induced by LEN. We did not monitor human cytomegalovirus (HCMV) replication but we cannot exclude that HCMV reactivation could explain these results, as HCMV is known to drive the expansion of NKG2C positive NK cells (24). When looking at antitumor functions, we noted a significant decrease in NK cell reactivity to K562 cells upon LEN treatment, irrespective of the parameter analyzed (Figure 2C; Figure S3A in Supplementary Material). This decrease correlated with a progressive decrease in NKp30 expression, itself associated with the gradual return to NK cell homeostasis (Figure 2C; Figure S3C in Supplementary Material). ADCC, which was already low before LEN therapy, was not improved by LEN treatment, despite a progressive increase in the percentage of CD16-expressing cells (Figure 2C; Figure S3C in Supplementary Material). Finally, not a single parameter showed clear-cut changes in expression upon discontinuation of LEN treatment, which clearly indicates the lack of LEN effect on peripheral NK cells, at least in this clinical setting.

## Discussion

Here, using a multiparametric approach, we demonstrated that induction therapy has a profound effect on NK cell maturation and activity. The long delay—about a year—before NK cells reached a quasi-normal maturation status was unexpected and resembles what is observed following Allogenic Stem Cell Transplantation (25) even though data in mouse models suggested a much faster kinetic following NK cell depletion (26). While NK cells repopulate the periphery more rapidly than T cells following myeloablation, their maturation and functions are restored much more slowly, similar to T cells.

Importantly, our data exclude a major stimulatory effect of LEN on NK cells when given alone. Indeed, we could not detect any signs of NK cell activation or any improvement of their functional capacity in patients undergoing several cycles of LEN treatment. This finding goes against a large number of articles describing a direct or indirect impact of LEN on NK cell activation. In particular, several articles reported a positive effect of LEN on NK cell-mediated antitumor activity *in vivo* in mouse models of transplanted tumors (27–29). Lioznov et al. showed that LEN leads to an increase in activated NK cells (NKp44^+^) but the effects were observed on few circulating NK cells (less than 5%) and no other activation signs nor any increase in NK cell function were described in this work (18). Our study is the first one to directly test the impact of LEN on multiple parameters of NK cell activation and function, longitudinally in human patients undergoing maintenance therapy with LEN alone. The absence of LEN effects on NK cells in our study correlates with the reported absence of protection conferred by LEN against infections by herpesviruses or against second primary cancers in MM patients (30, 31). As NK cells are major players of immune responses against cancer and infection by herpes viruses, a stimulatory effect of LEN could have led to some degree of protection. It is possible that NK cells from patients that underwent previous induction CTX are not responsive to LEN because of excessive damage on immune cells or on their environment. Induction cycles also include dexamethasone that has been shown to counter LEN effects on NK cells (32). However, all patients in this study started their LEN maintenance therapy at least 1 month after the end of induction CTX, which gave some time for them to recover from it and also to be cleared of dexamethasone. We therefore propose that LEN is not by itself sufficient to stimulate NK cell activity in patients and that it should be combined with more potent and directly stimulating drugs. Among potential candidates, ALT-803, an IL-15 derivative or the H9 IL-2 derivative, show very interesting results in different mouse models (33–35) and more recently in human (36). The synergy between LEN and IL-2 on NK cell activity *in vitro* (16) also supports this combination. As IL-2 and IL-15 activate commons signaling pathways (37), it would also be interesting to combine LEN with IL-15 or its derivatives, which have potent effects on NK cell compartment *in vivo* (38, 39).

Immumodulatory antibodies targeting immune checkpoints (40) and anti-KIR antibodies could also improve the LEN effects on NK cells. A Phase I Trial of the Anti-KIR Antibody IPH2101 and LEN was reported in Patients with Relapsed/Refractory MM (41) but the result of this trial is not published yet. This was based on preclinical data in a mouse model showing the efficacy of a LEN/anti-Ly49C combination (42).

In conclusion, we performed the first exhaustive monitoring of NK cell activity in patients taking LEN as single therapy. Our results unambiguously show a lack of stimulatory effect of LEN on peripheral NK cell activity. These results caution against the use of LEN as single-immunomodulatory therapy to improve NK cell activity in patients with cancer and provide a rationale to combine LEN with other drugs such as cytokines or monoclonal antibodies for this purpose.

## Ethics Statement

The clinical trial protocol was initially approved by the institutional ethics committee at the coordinating center (Purpan Hospital, Toulouse, France). For our study, patients provided an additional written informed consent in accordance with the declaration of Helsinki.

## Author Contributions

LAB, EC, and LUB performed the experiments. LK and GS recruited patients. OA performed statistical analysis. AM, PR, and CL have participated in the design of the experiments and wrote the manuscript. MA was in charge of IFM/DFCI protocol 2009. SV and TW have participated in the design of the experiments, the data analysis, and wrote the manuscript.

## Conflict of Interest Statement

The authors declare that the research was conducted in the absence of any commercial or financial relationships that could be construed as a potential conflict of interest.

## Supplementary Material

Figure S1 Representative gating strategy for the identification of peripheral blood natural killer (NK) cells. After exclusion of doublets and CD4^+^, CD14^+^, and CD19^+^ cells, NK cells were defined as CD3^−^ CD56^+^ lymphocytes **(A)**. Representative FACS plot of surface receptors **(B)**, activation markers **(C)**, and antitumor function parameters **(D)** analyzed.

Figure S2 Additional data for Figure 1: induction CTX impairs natural killer (NK) cell maturation and ADCC functions. Flow cytometry analysis of the indicated parameters in peripheral NK cells from HV and multiple myeloma patients after induction/consolidation CTX [post-Velcade Revlimid Dexamethasone (VRD)] (black dots are patients who received VRD, blue dots are patients who received stem-cell transplantation). Parameters were clustered in functional categories “antitumor function” **(A)**, “activation markers” **(B)**, and “cell surface receptors” **(C)**.

Figure S3 Additional data for Figure 2: lenalidomide (LEN) treatment neither activates natural killer (NK) cells nor improves their effector functions. Flow cytometry analysis of the indicated parameters in NK cells from patients monitored at different time-points before, during, or after LEN therapy. Charts of the percentages or MFI of indicated parameter within gated NK cells. Each line corresponds to one patient (black dots are patients who received Velcade Revlimid Dexamethasone, blue dots are patients who received stem-cell transplantation). Parameters were clustered in functional categories “antitumor function” **(A)**, “activation markers” **(B)**, and “cell surface receptors” **(C)**.
