# Supplementary figures and images for: One-Year Follow-Up of Natural Killer Cell Activity in Multiple Myeloma Patients Treated With Adjuvant Lenalidomide Therapy
Source: Front Immunol. 2018 Apr 13;9:704. doi: 10.3389/fimmu.2018.00704 (PMC5908898; doi:10.3389/fimmu.2018.00704)

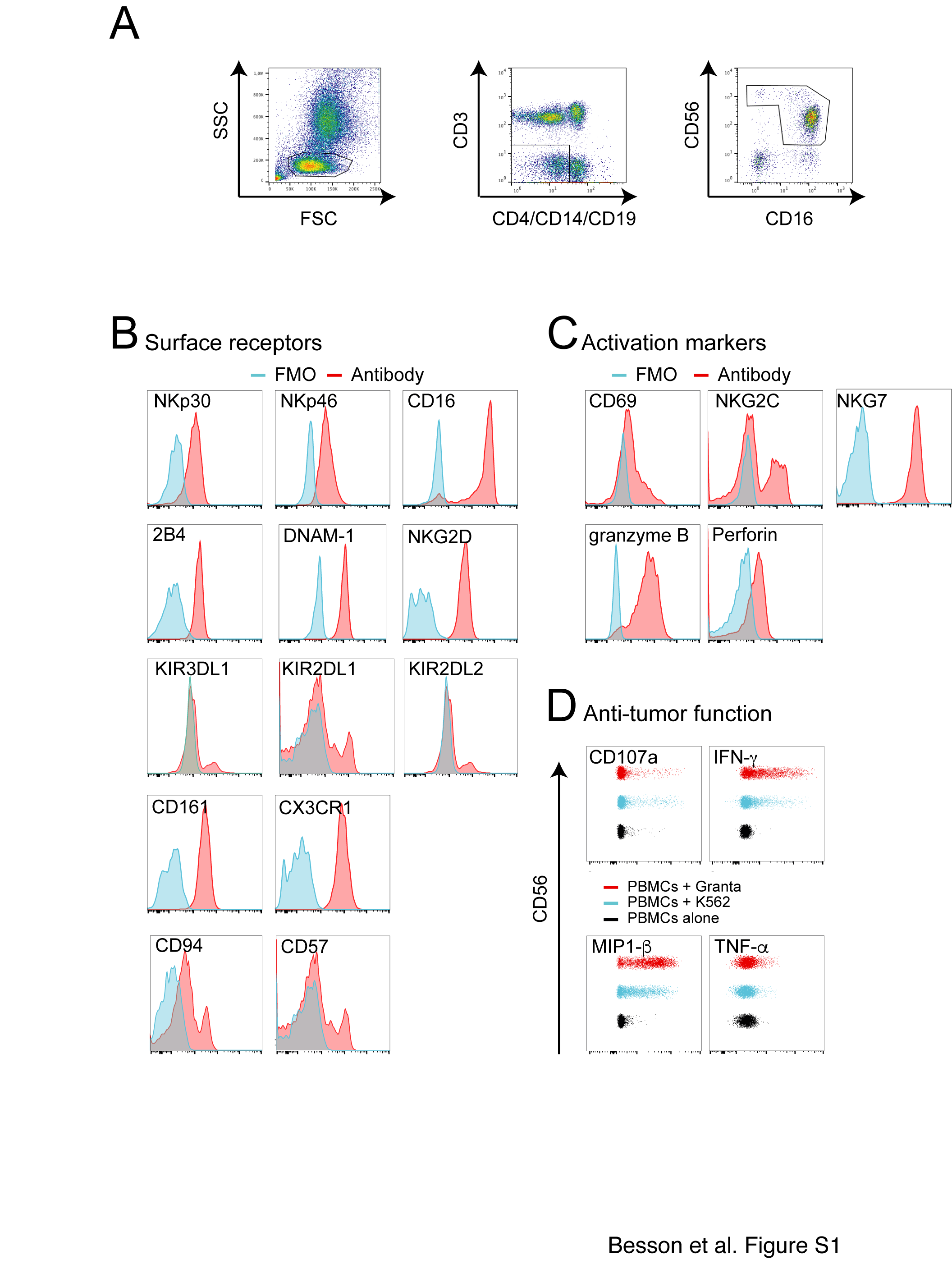

Supplement: Figure S1 — Representative gating strategy for the identification of peripheral blood natural killer (NK) cells. After exclusion of doublets and CD4+, CD14+, and CD19+ cells, NK cells were defined as CD3− CD56+ lymphocytes (A). Representative FACS plot of surface receptors (B), activation markers (C), and antitumor function parameters (D) analyzed. [file image_1.tif]

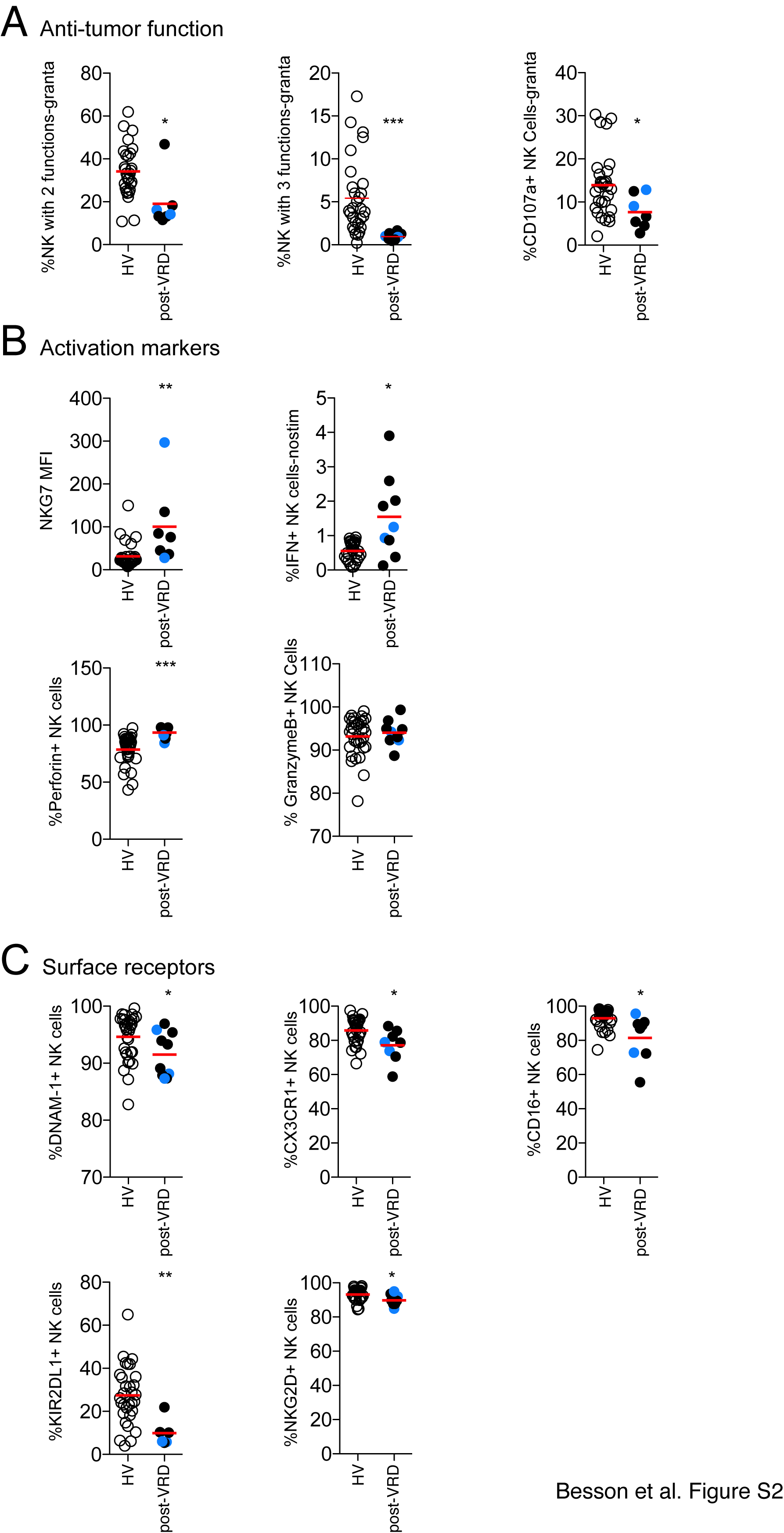

Supplement: Figure S2 — Additional data for Figure 1: induction CTX impairs natural killer (NK) cell maturation and ADCC functions. Flow cytometry analysis of the indicated parameters in peripheral NK cells from HV and multiple myeloma patients after induction/consolidation CTX [post-Velcade Revlimid Dexamethasone (VRD)] (black dots are patients who received VRD, blue dots are patients who received stem-cell transplantation). Parameters were clustered in functional categories “antitumor function” (A), “activation markers” (B), and “cell surface receptors” (C). [file image_2.tif]

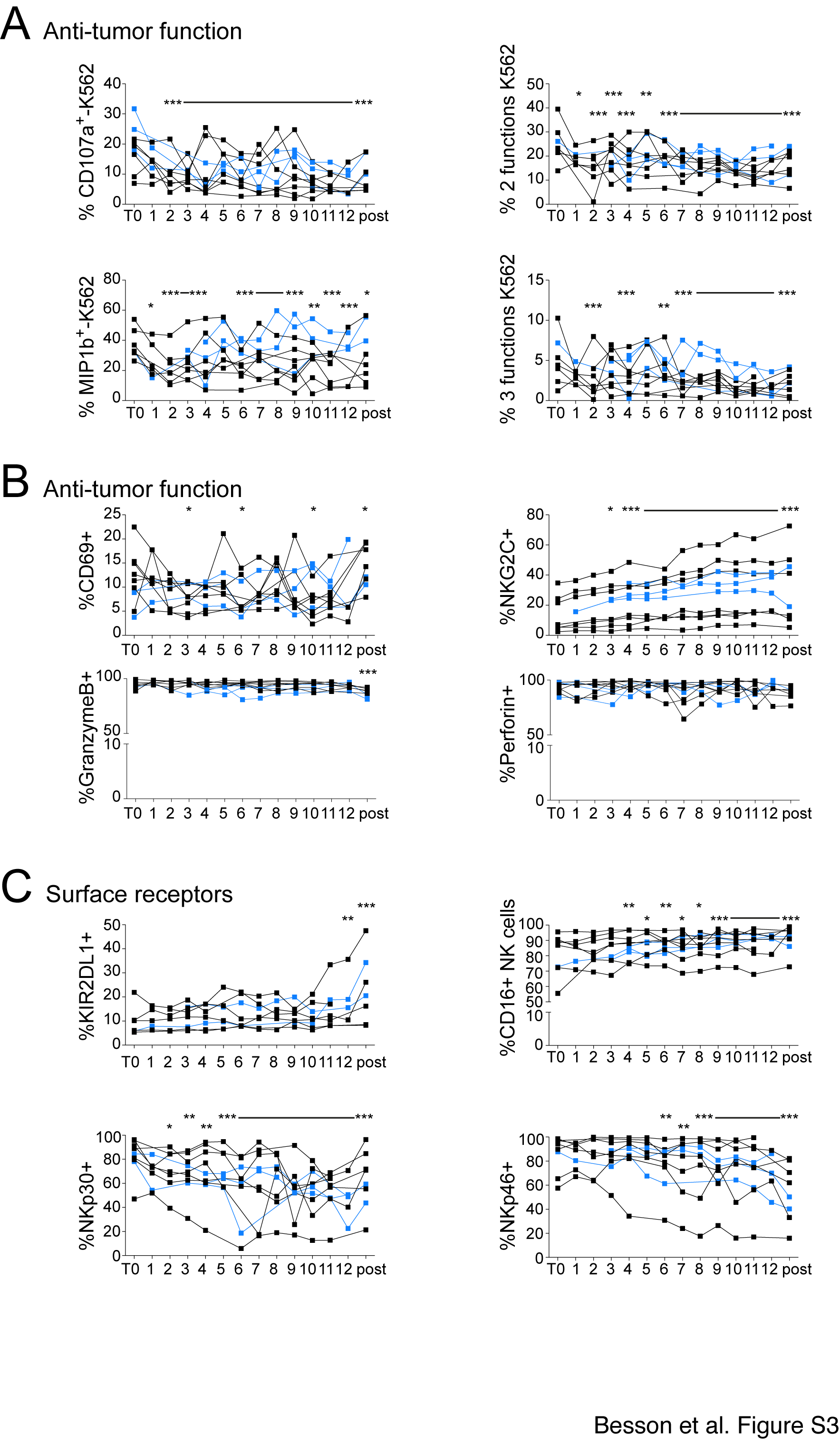

Supplement: Figure S3 — Additional data for Figure 2: lenalidomide (LEN) treatment neither activates natural killer (NK) cells nor improves their effector functions. Flow cytometry analysis of the indicated parameters in NK cells from patients monitored at different time-points before, during, or after LEN therapy. Charts of the percentages or MFI of indicated parameter within gated NK cells. Each line corresponds to one patient (black dots are patients who received Velcade Revlimid Dexamethasone, blue dots are patients who received stem-cell transplantation). Parameters were clustered in functional categories “antitumor function” (A), “activation markers” (B), and “cell surface receptors” (C). [file image_3.tif]
